# Impact of volume indices in bioelectrical impedance measurement on the assessment of cardiac function indices by echocardiography in hemodialysis patients

**DOI:** 10.1080/0886022X.2024.2375103

**Published:** 2024-07-08

**Authors:** Yudan Wang, Yang Wang, Shun Wu, Na Xu, Zhaoyong Zhang, Zhenmin Ruan, Rui Wang, Xin Geng, Chuanzhen Zhang, Zhiyong Luan, Guofang Chen, Hongqi Ren

**Affiliations:** a Department of Nephrology, The Affiliated Huaihai Hospital of Xuzhou Medical University, Xuzhou, Jiangsu, China; b Department of Ultrasound, The Affiliated Huaihai Hospital of Xuzhou Medical University, Xuzhou, Jiangsu, China; c Department of Neurology, The Affiliated Xuzhou Central Hospital of Xuzhou Medical University, Xuzhou, Jiangsu, China

**Keywords:** Hemodialysis, volume indices, bioelectrical impedance, cardiac function, echocardiography

## Abstract

**Introduction:**

Cardiovascular events resulting from volume overload are a primary cause of mortality in hemodialysis patients. Bioelectrical impedance analysis (BIA) is significantly valuable for assessing the volume status of hemodialysis (HD) patients. In this article, we explore the correlation between the volume index measured by BIA and the cardiac function index assessed by echocardiography (ECG) in HD patients.

**Methods:**

Between April and November 2018, we conducted a cross-sectional study involving randomly selected 126 maintenance HD patients. Comprehensive data on medical history and laboratory test results were collected. Subsequently, we investigated the correlation between volume indices measured by BIA and cardiac function parameters by ECG.

**Results:**

We discovered a significant correlation between the volume indices measured by BIA and various parameter of cardiac function. The Left Ventricular Hypertrophy (LVH) group exhibited higher levels of the percentage of Extracellular Water (ECW%) and the percentage of Total Body Water (TBW%) compared to the Non-LVH group. Extracellular Water (ECW) and Third Interstitial Fluid Volume (TSFV) were identified as independent risk factors for Left Ventricular Mass (LVM), and both demonstrated a high predictive value for LVM. ECW% emerged as an independent risk factor for the Left Ventricular Mass Index (LVMI), with a high predictive value for LVMI.

**Conclusion:**

ECW and TSFV were found to be positively associated with cardiac function parameters in HD patients.

## 1. Introduction

Cardiovascular events (CVE) are the primary cause of mortality in hemodialysis patients, responsible for 40% of deaths according to the latest statistics from the US Renal Data System [1]. The cardiovascular mortality rate is 9-fold higher in these patients compared with age- and sex-matched individuals in the general population [2]. Volume overload is a significant contributor to this elevated mortality risk and is a prevalent complication in dialysis patients. It has been linked to hypertension, arterial stiffness, left ventricular hypertrophy, and congestive heart failure [3,4]. Therefore, the assessment and management of volume status play a crucial role in the care of hemodialysis patients [5]. Early identification of cardiac function abnormalities and prompt, aggressive treatment to alleviate symptoms are essential components of end-stage renal disease (ESRD) patient management [6].

Traditional methods of volume evaluation rely on clinical experience and symptoms to assess volume status, identifying either volume overload or underload [7,8]. However, these conventional methods may lack sensitivity to detect subtle changes in volume, which can present challenges in the timely prevention and management of cardiovascular complications [9]. Bioelectrical impedance is a simple, noninvasive method for evaluating volume at a low cost and with adequate repeatability, which has emerged as a promising solution to this challenge [10]. Several studies have demonstrated the utility of multi-frequency bioimpedance in aiding the clinical assessment and management of volume status in dialysis patients [11,12]. In addition, our previous research has also demonstrated that adjusting dry weight with guidance from multi-frequency bioimpedance can effectively alleviate volume overload and improve blood pressure control in dialysis patients [13]. Furthermore, we further used BIA and echocardiography in combination to investigate the correlation between post-dialysis volume state and intra-dialytic hypertension(IDH), the results showed that post-dialysis volume expansion is an important factor for the development of IDH [14].

Echocardiography (ECG) findings have proven to be reliable and are important noninvasive tools for assessing the intravascular compartment volume load in ESRD patients [15]. Regarding the relationship between volume indices measured by Bioelectrical Impedance Analysis (BIA) and cardiac function indices obtained through ECG, several studies have shown that both can evaluate dry weight and even predict mortality in dialysis patients [16,17]. However, there is no definitive conclusion about the precise relationship between parameters measured by BIA and ECG.

In this manuscript, we explore the potential of volume indices obtained through bioelectrical impedance, such as Total Segmental Fluid Volume (TSFV), Extracellular Water (ECW), ECW%, Total Body Water (TBW), TBW%, Intracellular Water (ICW) and the percentage of Intracellular Water (ICW%) to assess cardiac function indices in patients undergoing maintenance hemodialysis.

## 2. Information and methods

### 2.1. Study population

Between April 2018 and November 2018, we randomly selected 126 patients undergoing stable hemodialysis in the Huaihai Hospital affiliated to Xuzhou Medical University. Inclusion criteria comprised an age range of 18 to 75 years and a minimum of six months of regular hemodialysis. Exclusion criteria included patients with an expected survival time of fewer than six months, diagnosed malignancy, inability to undergo bioelectrical impedance testing (e.g., cardiovascular stenting, pacemaker implantation, joint replacement, amputation, severe peripheral vascular disease), history of surgery, trauma, or severe infection within the past three months, pregnancy or lactation, and patient refusal. This study was reviewed along with endorsed by the Huaihai Hospital Medical Ethics Board, Xuzhou Medical University (Ethics No. 2018-007).

### 2.2. Research program

#### 2.2.1. Observed indices

Clinical data from the 126 patients receiving maintenance hemodialysis were collected, including gender, age, duration of dialysis, dialysis mode, pre-dialysis weight, post-dialysis weight, water loss, pre-dialysis systolic blood pressure (SBP), pre-dialysis diastolic blood pressure (DBP), post-dialysis SBP, and post-dialysis DBP. Bioelectrical impedance indices (ECW, ICW, TBW, TSFV, lean body mass),and laboratory parameters (blood urea nitrogen, serum creatinine, potassium ion, sodium ion, chloride ion, calcium ion, fasting blood collection for routine blood tests, liver function, plasma albumin, lipids, whole segment parathyroid hormone) were also recorded.

#### 2.2.2. Measurement of relevant indices

##### 2.2.2.1. Blood pressure measurement

Blood pressure was measured using The Braun Dialog electronic sphygmomanometer and the Omron HEM-4030 electronic sphygmomanometer to measure blood pressure on the non-fistula side of the upper arm. Measurements were taken before and after dialysis.

##### 2.2.2.2. Whole-Body multi-frequency bioelectrical impedance testing

A multi-frequency bioelectrical impedance analysis device (Bodystat QuadScan 4000, UK) was used one hour before dialysis at the time of their first session of the week. Patients were in the supine position, and electrodes were placed on the forearm and ankle on the non-fistula side. Resistances and reactances were measured under discrete frequencies of 5, 50, 100, and 200 kHz. ECW, ECW%, ICW, ICW%, TBW, TBW% and TSFV were calculated automatically by the Body Composition Monitor software. Measurements were taken by two trained physicians who were blinded to other clinical data of the patient.

##### 2.2.2.3. Echocardiography

Transthoracic color echocardiography was performed using a Philips iE33 color echocardiography diagnostic instrument with a probe frequency of 1.0–5.0 MHz (Philips Ultrasound, Inc. Bothell, WA, USA). The examinations were performed one hour before dialysis and according to the American Society of Color Echocardiography Guidelines [18]. The images were evaluated in terms of cardiac structure and function by an experienced ultrasonic diagnostic physician blinded to other clinical data of the patient. Cardiac ultrasound evidence, including LVEF, E, A, left ventricular mass (LVM), left ventricular volume (LVV), left atrial volume (LAV), posterior wall thickness at end-diastole (PWTD), posterior wall thickness at end-systole (PWTs), left ventricular end-diastolic diameter (LVDD), left ventricular end-systolic diameter (LVDS), interven­tricular septal thickness in diastole (IVSTD) and interventricular septal thickness in systole (IVSTs) was collected. E/A and left ventricular mass index (LVMI) were calculated.

##### 2.2.2.4. Grouping

LVMI was calculated as LVM/BSA. Patients were grouped based on LVMI, with LVMI > 95 g/m^2^ for women and >115 g/m^2^ for men classified as the LVH group and LVMI ≤ 95 g/m^2^ for women and ≤115 g/m^2^ for men as the non-LVH group, following the definition of LVH [19]. The boundary value of LVM was fixed at 210 g, as per Park et al. [20].

### 2.3. Statistical methods

Statistical analysis was performed using SPSS 26.0 software. Normally distributed measurements were expressed as the mean ± standard deviation, and comparisons were made using paired samples t-test for two groups and one-way analysis of variance for three or more groups. Non-normally distributed data were expressed as median and quartile distance, and the non-parametric rank sum test was used for comparisons between two groups. Risk factors for abnormal cardiac function indices were analyzed using binary logistic regression. The ROC method was employed for predicting cardiac function abnormalities. All tests were two-sided, with a statistical significance set at *p* < 0.05.

## 3. Results

### 3.1. General information

126 patients were enrolled in the study. The enrolled patients included 77 males (61.1%) and 49 females (38.9%), with an average age of 49.6 ± 11.5 years (range 24∼72 years). The most common diagnosis was primary glomerular disease, observed in 50 patients (39.7%), followed by hypertensive renal impairment in 16 patients (12.7%), hereditary nephropathy in 11 patients (8.73%), diabetic nephropathy in 10 patients (7.93%), lupus nephritis in 7 patients (5.56%), interstitial nephritis in 4 patients (3.17%), and unknown conditions in 28 patients (22.2%).

### 3.2. Laboratory examination

Among all participants, mean dialysis duration was 5.95 ± 4.82 years, pre-dialysis SBP was 144.9 ± 19.5 mmHg, pre-dialysis DBP was 87.1 ± 12.8 mmHg, hemoglobin level was 120.3 ± 20.6 g/L, ALB was 47.2 ± 3.51 g/L. After further analysis, it was found that pre-dialysis SBP and DBP were higher in the LVH group than in the non-LVH group(*p* < 0.05), and the levels of 25(OH)_2_D was significantly higher in the LVH group than in the non-LVH group (29.2 ± 11.45 vs 25.0 ± 9.13 ng/mL, *p* = 0.031). The other indices such as BUN, SCr, UA, Ca^2+^, P^3-^, Ca × P, iPTH, GLU, ALB, TCH, TG, HDL, LDL and HsCRP showed no significant difference among the Groups (Table 1).

**Table 1.** General information and laboratory parameter values for the patients.

	All patients (*N* = 126)	Non-LVH (*N* = 79)	LVH (*N* = 47)	P-value
Gender(M/F)	77/49	43/36	34/13	0.081
Age (y)	49.60 ± 11.5	49.03 ± 11.4	50.19 ± 12.0	0.601
Dialysis duration (y)	5.95 ± 4.82	6.18 ± 5.22	5.95 ± 4.15	0.807
Pre-dialysis Weight (kg)	62.0 ± 10.4	62.3 ± 10.8	61.14 ± 10.0	0.569
Post-dialysis Weight (kg)	59.8 ± 10.2	60.1 ± 10.7	58.9 ± 9.83	0.535
Pre-dialysis SBP (mmHg)	144.9 ± 19.5	141.0 ± 19.1	151.43 ± 18.5	0.005
Pre-dialysis DBP (mmHg)	87.1 ± 12.8	85.2 ± 12.2	90.0 ± 13.5	0.049
Hb(g/L)	120.3 ± 20.6	120.5 ± 19.1	120.8 ± 23.6	0.954
BUN(mg/dl)	70.1 ± 15.9	68.2 ± 16.9	73.5 ± 13.9	0.085
SCr(mg/dl)	10.2 ± 3.9	10.0 ± 3.56	10.55 ± 4.72	0.487
UA(μmol/L)	347.1 ± 92.0	346.16 ± 93.5	341.8 ± 92.5	0.805
Ca^2+^(mmol/L)	2.47 ± 0.21	2.47 ± 0.19	2.44 ± 0.23	0.394
P^3-^(mmol/L)	1.65 ± 0.45	1.68 ± 0.46	1.67 ± 0.47	0.798
Ca*P(mg^2^/dl^2^)	50.7 ± 15.0	50.7 ± 14.6	50.9 ± 16.3	0.938
lgiPTH	2.59 ± 0.42	2.58 ± 0.38	2.61 ± 0.47	0.216
25(OH)_2_D(ng/ml)	26.1 ± 10.2	25.0 ± 9.13	29.2 ± 11.5	0.031
GLU(mmol/L)	5.34 ± 2.37	5.22 ± 1.05	5.6 ± 3.85	0.418
ALB (g/L)	47.2 ± 3.51	47.2 ± 3.57	47.4 ± 3.56	0.819
TCH(mmol/L)	4.78 ± 0.99	4.86 ± 1.03	4.66 ± 0.89	0.289
TG(mmol/L)	1.69 ± 0.88	1.78 ± 1.01	1.51 ± 0.54	0.109
HDL(mmol/L)	0.86 ± 0.30	0.89 ± 0.30	0.85 ± 0.29	0.884
LDL(mmol/L)	2.49 ± 0.73	2.50 ± 0.73	2.49 ± 0.71	0.967
HsCRP (μg/ml)	3.4 ± 3.65	3.56 ± 2.89	3.15 ± 4.87	0.562

Abbreviations: HB: hemoglobin; BUN: Blood urea nitrogen; SCr: serum creatinine; UA: uric acid; Ca: Calcium; P: Phosphorus; PTH: Parathyroid hormone; GLU: glucose; ALB: albumin; TCH: total cholesterol; TG: triglyceride; HDL: high-density lipoprotein; LDL: Low-density lipoprotein; HsCRP: High-sensitivity c-reactive protein.

### 3.3. Comparison of cardiac function parameters grouped by trichotomy according to ECW

**Figure 1.**
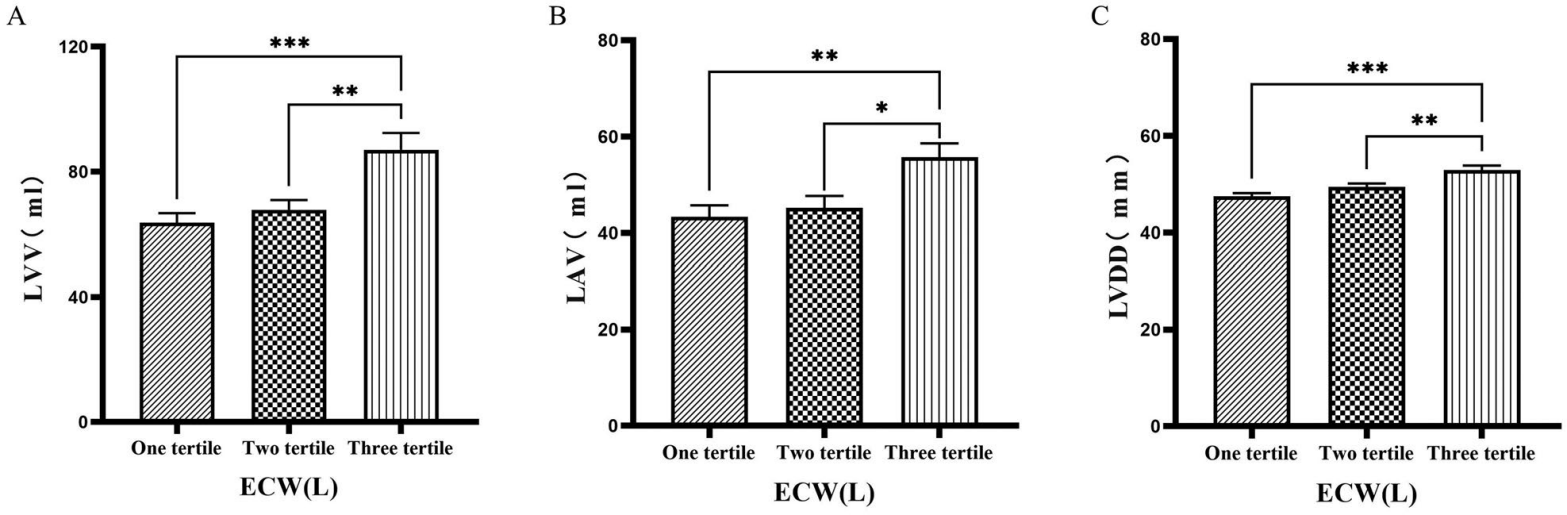
Comparison of cardiac function indices for different ECW groupings. 126 patients were grouped into tertiary groups according to ECW (first tertiary group: ECW ≤ 14.2 L, second tertiary group: 14.2 L < ECW ≤ 16.7 L, third tertiary group: ECW > 16.7 L). LVV(A), LAV(B), LVDD(C), of the patients was compared in three different ECW groupings. The data was analyzed using one-way analysis of variance. *P < 0.05; **P < 0.01; ***P < 0.001. LVV: left ventricular volume; LAV: left atrial volume; LVDD: left ventricular end-diastolic diameter; ECW: extracellular water.

**Figure 2.**
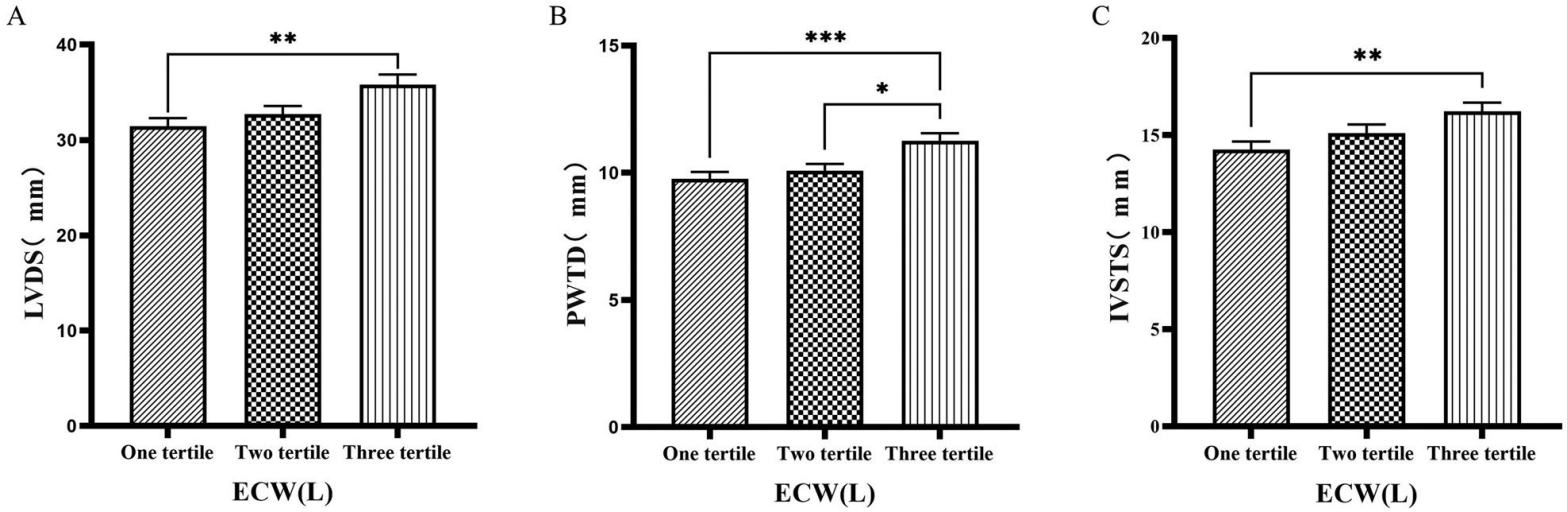
Comparison of cardiac function indices for different ECW groupings. 126 patients were grouped into tertiary groups according to ECW (first tertiary group: ECW ≤ 14.2 L, second tertiary group: 14.2 L < ECW ≤ 16.7 L, third tertiary group: ECW > 16.7 L). LVDs(A), PWTD(B), IVSTs(C) of the patients was compared in three different ECW groupings. The data was analyzed using one-way analysis of variance. *P < 0.05; **P < 0.01; ***P < 0.001. LVDs: left ventricular end-systolic diameter; PWTD: posterior wall thickness at end-diastole; IVSTS: interventricular septal thickness in systole; ECW: extracellular water.

The 126 patients were categorized into tertile groups based on ECW. The levels of LVV, LAV, LVDD, PWTD, LVDs, and IVSTs in the third tertile group (ECW > 16.7 L) were significantly greater than those in the first tertile group (ECW ≤ 14.2 L), (*p* < 0.05) (Figures 1 & 2).

### 3.4. Comparison of cardiac function parameters grouped by quartiles according to ECW%

**Figure 3.**
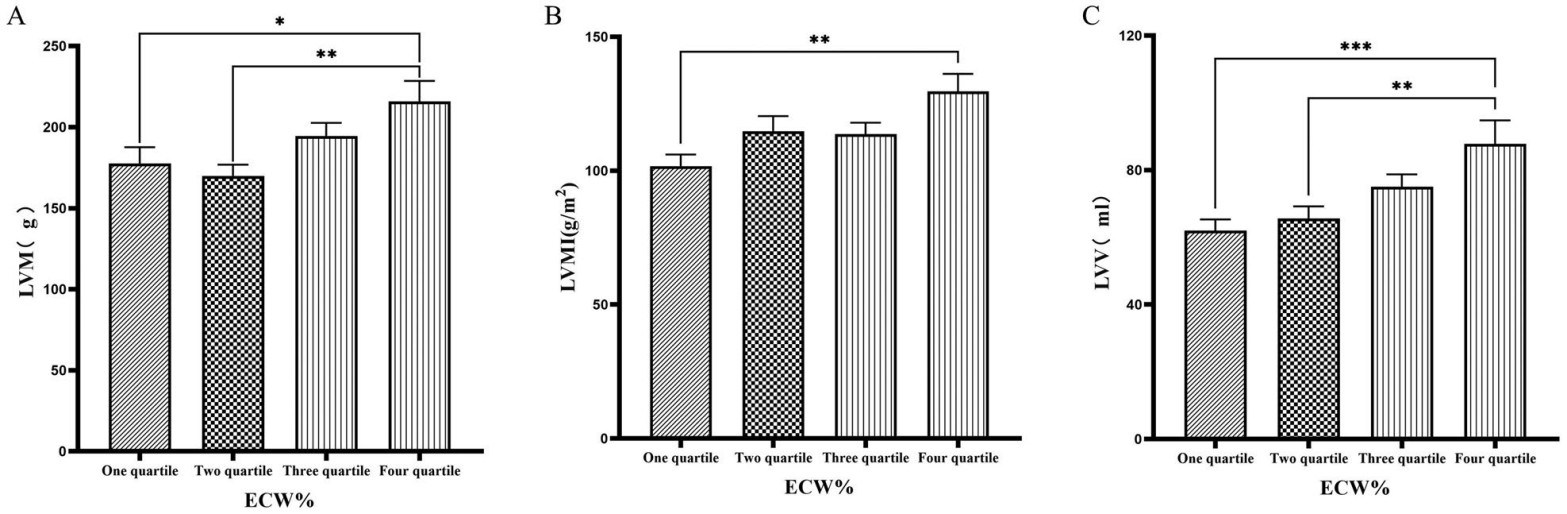
Comparison of cardiac function indices for different ECW% groupings. 126 patients were grouped into quartiles groups according to ECW% (first tertiary group: ECW% ≤ 23.175%, second quartile group: 23.175% < ECW% ≤ 25.450%, third quartile group: 25.450% < ECW% ≤ 27.1%). LVM(A), LVMI(B), LVV(C), of the patients was compared in four different TBW groupings. The data was analyzed using one-way analysis of variance. *P < 0.05; **P < 0.01; ***P < 0.001. LVM: left ventricular mass; LVMI: left ventricular mass index; LVV: left ventricular volume; ECW%: the percent of extracellular water.

**Figure 4.**
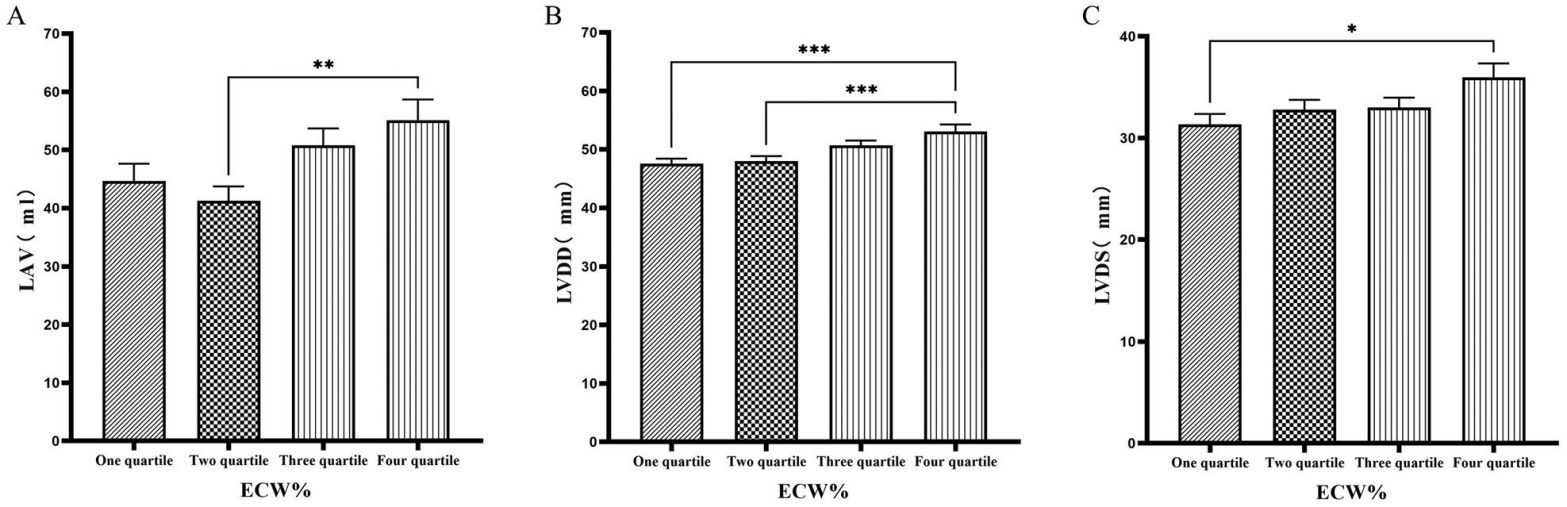
Comparison of cardiac function indices for different ECW% groupings. 126 patients were grouped into quartiles groups according to ECW% (first tertiary group: ECW% ≤ 23.175%, second quartile group: 23.175% < ECW% ≤ 25.450%, third quartile group: 25.450% < ECW% ≤ 27.1%). LAV(A), LVDD (B), LVDs(C) of the patients was compared in four different TBW groupings. The data was analyzed using one-way analysis of variance. *P < 0.05; **P < 0.01; ***P < 0.001. LAV: left atrial volume; LVDD: left ventricular end-diastolic diameter; LVDs: left ventricular end-systolic diameter; ECW%: the percent of extracellular water.

Patients were grouped into quartiles based on ECW%. The fourth quartile group (ECW% > 27.1%) exhibited significantly higher values of LVM, LVMI, LVV, LAV, LVDD, and LVDs compared to those in the first quartile group (ECW% ≤ 23.175%) (*p* < 0.05) (Figures 3 & 4).

### 3.5. Comparison of cardiac function parameters grouped by trichotomy according to TSFV

**Figure 5.**
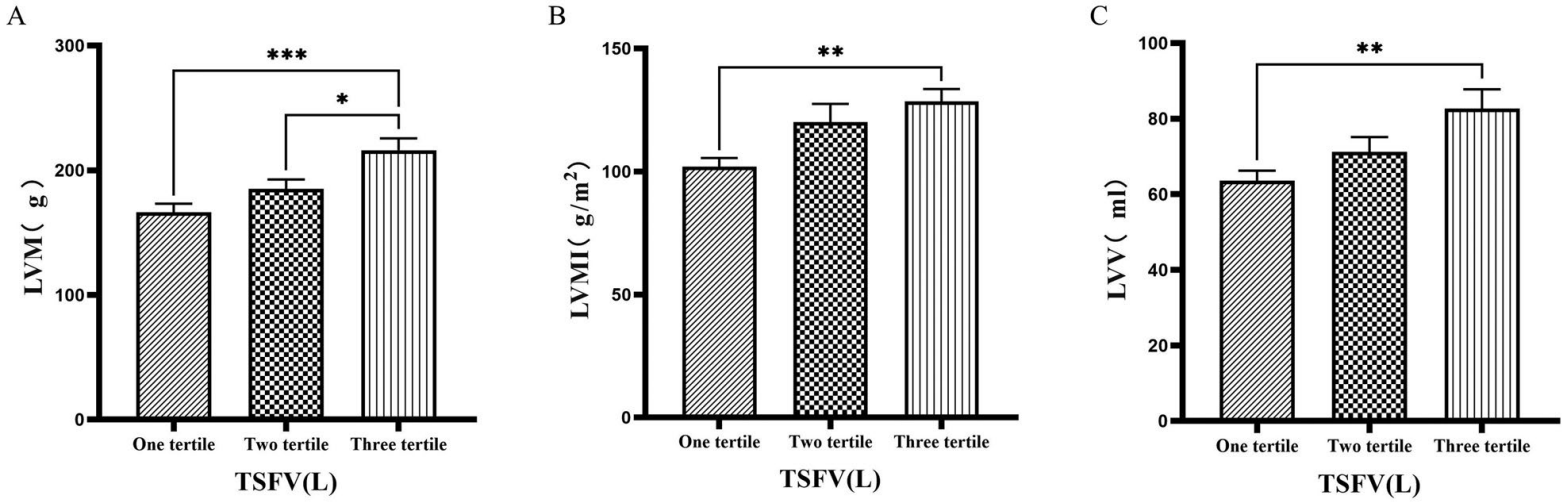
Comparison of cardiac function indices for different TSFV subgroups. 126 patients were grouped into tertiary groups according to TSFV (first tertiary group: TSFV ≤ 2.933 L, second tertiary group: 2.933 L < TSFV volume ≤ 3.967 L, third tertiary group: TSFV > 3.967 L). LVM(A), LVMI(B), LVV(C) of the patients was compared in three different TSFV groupings. The data was analyzed using one-way analysis of variance. *P < 0.05; **P < 0.01; ***P < 0.001. LVM: left ventricular mass; LVMI: left ventricular mass index; LVV: left ventricular volume; TSFV: third interstitial fluid volume.

**Figure 6.**
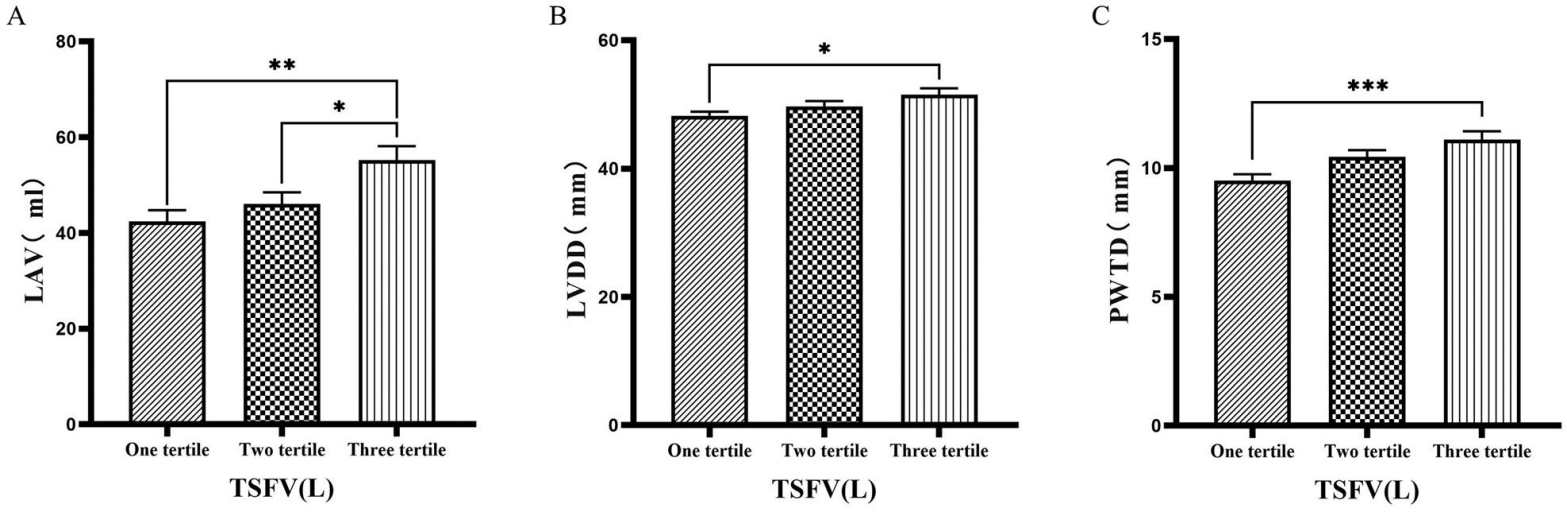
Comparison of cardiac function indices for different TSFV subgroups. 126 patients were grouped into tertiary groups according to TSFV (first tertiary group: TSFV ≤ 2.933 L, second tertiary group: 2.933 L < TSFV volume ≤ 3.967 L, third tertiary group: TSFV > 3.967 L). LAV(A), LVDD(B), PWTD(C) of the patients was compared in three different TSFV groupings. The data was analyzed using one-way analysis of variance. *P < 0.05; **P < 0.01; ***P < 0.001. LAV: left atrial volume; LVDD: left ventricular end-diastolic diameter; PWTD: posterior wall thickness at end-diastole. TSFV: third interstitial fluid volume.

**Figure 7.**
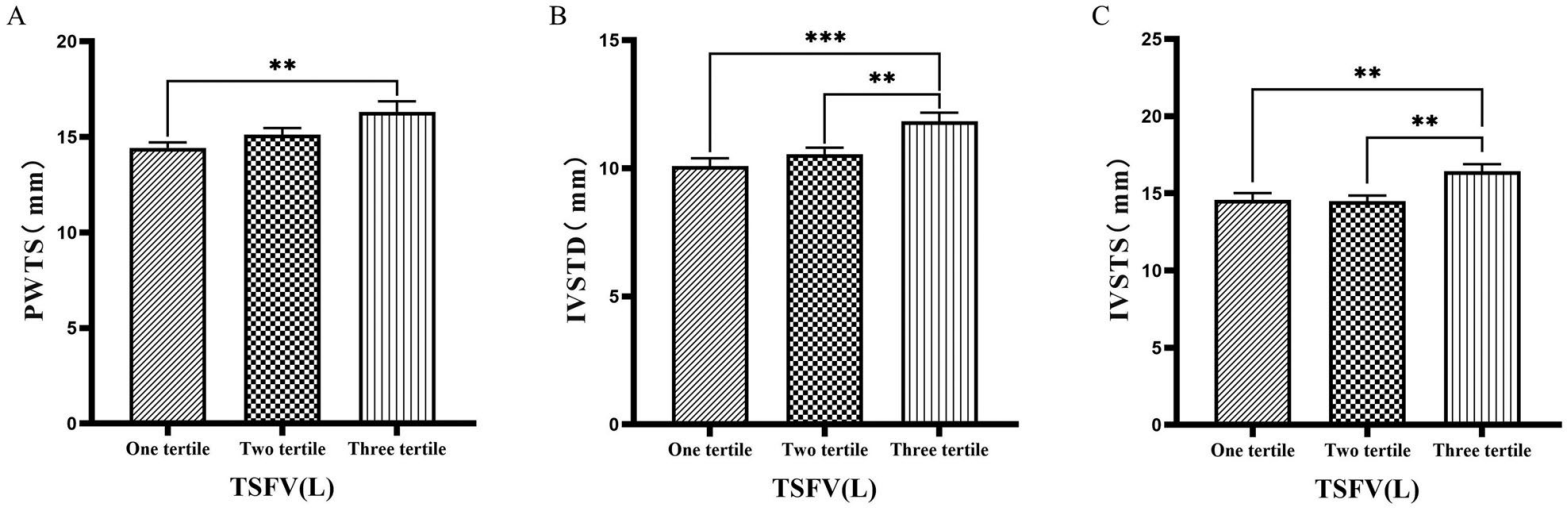
Comparison of cardiac function indices for different TSFV subgroups. 126 patients were grouped into tertiary groups according to TSFV (first tertiary group: TSFV ≤ 2.933 L, second tertiary group: 2.933 L < TSFV volume ≤ 3.967 L, third tertiary group: TSFV > 3.967 L). PWTs(A), IVSTD(B), IVSTS(C) of the patients was compared in three different TSFV groupings. The data was analyzed using one-way analysis of variance. *P < 0.05; **P < 0.01; ***P < 0.001. PWTs: posterior wall thickness at end-systole; IVSTD: end-diastolic septal thickness; IVSTS: interventricular septal thickness in systole; TSFV: third interstitial fluid volume.

Grouping based on TSFV revealed that the third tertile group (TSFV > 3.967 L) had significantly greater values of LVM, LVMI, LVV, LAV, IVSTD, LVDD, PWTD, IVSTs, and PWTs compared to those in the first tertile group (TSFV ≤ 2.933 L)(*p* < 0.05) (Figures 5–7).

### 3.6. Comparison of cardiac function parameters grouped by trichotomy according to TBW

**Figure 8.**
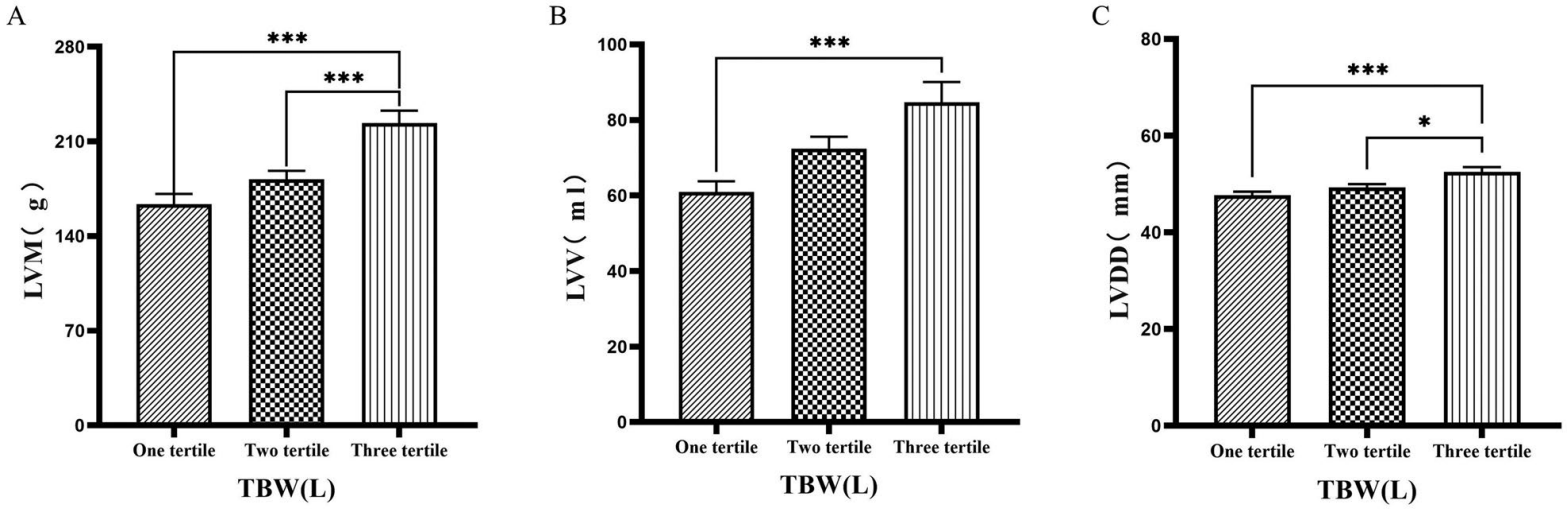
Comparison of cardiac function indices for different TBW subgroups. 126 patients were grouped into tertiary groups according to TBW (first tertiary group: TBW ≤ 27.9 L, second tertiary group: 27.9 L < TBW ≤ 34.3 L, third tertiary group: TBW > 34.3 L). LVM(A), LVV(B), LVDD(C) of the patients was compared in three different TBW groupings. The data was analyzed using one-way analysis of variance. *P < 0.05; **P < 0.01; ***P < 0.001. LVM: left ventricular mass; LVV: left ventricular volume; LVDD: left ventricular end-diastolic diameter; TBW: total body water.

**Figure 9.**
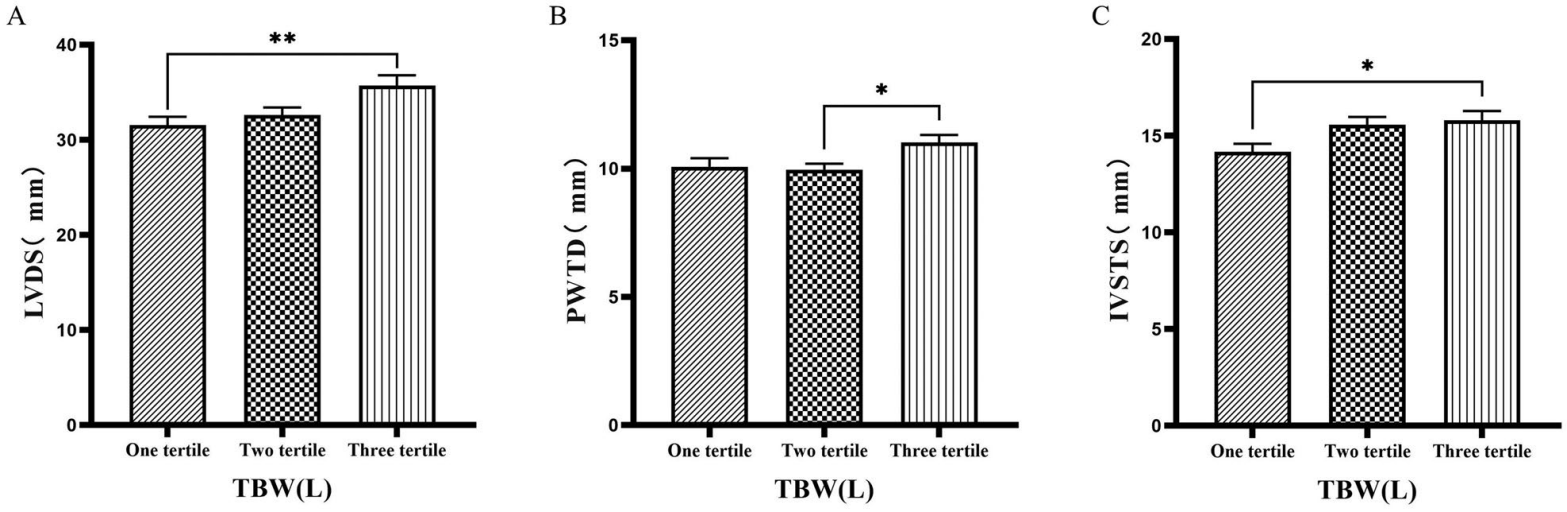
Comparison of cardiac function indices for different TBW subgroups. 126 patients were grouped into tertiary groups according to TBW (first tertiary group: TBW ≤ 27.9 L, second tertiary group: 27.9 L < TBW ≤ 34.3 L, third tertiary group: TBW > 34.3 L). LVDs(A), PWTD(B), IVSTS(C) of the patients was compared in three different TBW groupings. The data was analyzed using one-way analysis of variance. *P < 0.05; **P < 0.01; ***P < 0.001. LVDS: left ventricular end-systolic diameter; PWTD: posterior wall thickness at end-diastole; IVSTS: interventricular septal thickness in systole; TBW: total body water.

Patients were grouped into tertiles based on TBW. It was found that the third tertile group (TBW > 34.3 L) had significantly greater values of LVM, LVV, LVDD, PWTD, IVSTs, and LVDs compared to the first tertile group (TBW ≤ 27.9 L) (Figures 8 & 9).

### 3.7. Comparison of bioelectrical impedance indices between the LVH group and the non-LVH group

Patients were divided into left ventricular hypertrophy (LVH) and non-LVH groups. LVV, LAV, LVDD, PWTD, IVSTD, PWTs, IVSTs, ECW%, and TBW% were significantly higher in the LVH group than in the non-LVH group (*p* < 0.05), while other indices showed no significant differences (Table 2).

**Table 2.** Comparison of bioelectrical impedance indices and cardiac function indices between the LVH group and the non-LVH group.

	Non-LVH (*N* = 79)	LVH (*N* = 47)	P-value
ICW%	25.16 ± 4.62	26.55 ± 4.70	0.120
ICW(L)	15.57 ± 3.54	16.19 ± 3.50	0.358
ECW%	24.88 ± 2.60	26.11 ± 2.67	0.016
ECW(L)	15.39 ± 2.62	15.85 ± 2.32	0.346
TBW%	49.66 ± 7.19	52.65 ± 6.73	0.028
TBW(L)	30.72 ± 6.01	32.03 ± 5.55	0.243
ECW/ICW	101.16 ± 15.15	100.45 ± 15.01	0.807
Pre-dialysis weight (Kg)	62.29 ± 10.77	61.14 ± 9.99	0.569
Post-dialysis weight (Kg)	60.10 ± 10.67	58.85 ± 9.83	0.525
Water removal (Kg)	2.15 ± 0.92	2.26 ± 0.70	0.442
LVEF (%)	60.42 ± 5.58	58.74 ± 11.93	0.293
E (m/s)	71.25 ± 18.02	72.09 ± 20.67	0.817
E/A	0.93 ± 0.32	0.84 ± 0.33	0.151
LVV (ml)	61.78 ± 15.2	87.79 ± 26.55	0.001
LAV (ml)	41.54 ± 15.07	56.33 ± 14.05	0.001
LVDD (mm)	47.77 ± 4.29	52.38 ± 5.16	0.001
PWTD (mm)	9.59 ± 1.58	11.32 ± 1.61	0.001
IVSTD (mm)	10.23 ± 1.84	11.54 ± 1.94	0.001
IVSTs (mm)	14.46 ± 2.68	16.09 ± 2.81	0.002
LVDs (mm)	32.77 ± 6.11	33.77 ± 6.61	0.409
PWTs (mm)	14.56 ± 2.12	16.12 ± 3.07	0.001

Abbreviations: ECW: extracellular water; ECW%: the percent of extracellular water; ICW: intracellular water; ICW%: the percentage of intracellular water; TBW: total body water; TBW%: the percentage of total body water; ECW/ICW: Extracellular Water/Intracellular Water; LVV: left ventricular volume; LAV: left atrial volume; LVDD: left ventricular end-diastolic diameter; PWTD: posterior wall thickness at end-diastole; IVSTD: interventricular septal thickness in diastole; IVSTs: interventricular septal thickness in systole; LVDs: left ventricular end-systolic diameter; PWTs: posterior wall thickness at end-systole.

### 3.8. Regression analysis of factors influencing cardiac function parameters in maintenance hemodialysis patients

Logistic regression analysis identified ECW and TSFV were independent risk factors for LVM (*p* < 0.05). ECW% was an independent risk factor for LVMI and LVV (*p* < 0.05). While ECW was an independent risk factor for PWTD (*p* = 0.01). (Table 3).

**Table 3.** Logistic regression analysis of cardiac function indices.

	Factors	β	Wald	OR(95% CI)	P-value
LVM (g)	ECW	0.371	9.342	1.449 (1.142,1.838)	0.002
	TSFV	0.436	3.980	1.546 (1.008,2.373)	0.046
LVMI	ECW%	0.501	6.291	1.651 (1.116, 2.443)	0.012
	TSFV	−0.522	2.947	0.593 (0.327, 1.077)	0.086
LVV (ml)	ECW	−0.210	2.517	0.811(0.626,1.051)	0.113
	ECW%	−0.488	12.004	0.614(0.465,0.809)	0.001
LAV (ml)	ECW	−0.003	0.001	0.997(0.838,1.186)	0.970
LVEF (%)	ECW	−0.065	0.228	0.937(0.718,1.223)	0.633
E (m/s)	ECW/ICW	0.051	0.188	1.052(0.836,1.323)	0.665
E/A	ECW/ICW	−0.037	0.231	0.964(0.829,1.12)	0.631
PWTD (mm)	ECW	0.266	6.644	1.304(1.066,1.597)	0.010
LVDD (mm)	TSFV	−0.361	0.098	0.697(0.072,6.713)	0.755
	ECW/ICW	−0.075	0.907	0.927(0.794,1.083)	0.341
IVSTD (mm)	TSFV	−0.747	0.704	0.474(0.083,2.712)	0.401
	ECW/ICW	−0.09	1.995	0.914(0.806,1.036)	0.158

Abbreviations: LVM: left ventricular mass; LVMI: left ventricular mass index; LVV: left ventricular volume; PWTD: posterior wall thickness at end-diastole; LAV: left atrial volume; LVDD: left ventricular end-diastolic diameter; IVSTD: interventricular septal thickness in diastole ECW: extracellular water; ECW%: the percent of extracellular water; TSFV: third interstitial fluid volume.

ECW/ICW: Extracellular Water/Intracellular Water.

### 3.9. Predictive value of cardiac function parameters

**Figure 10.**
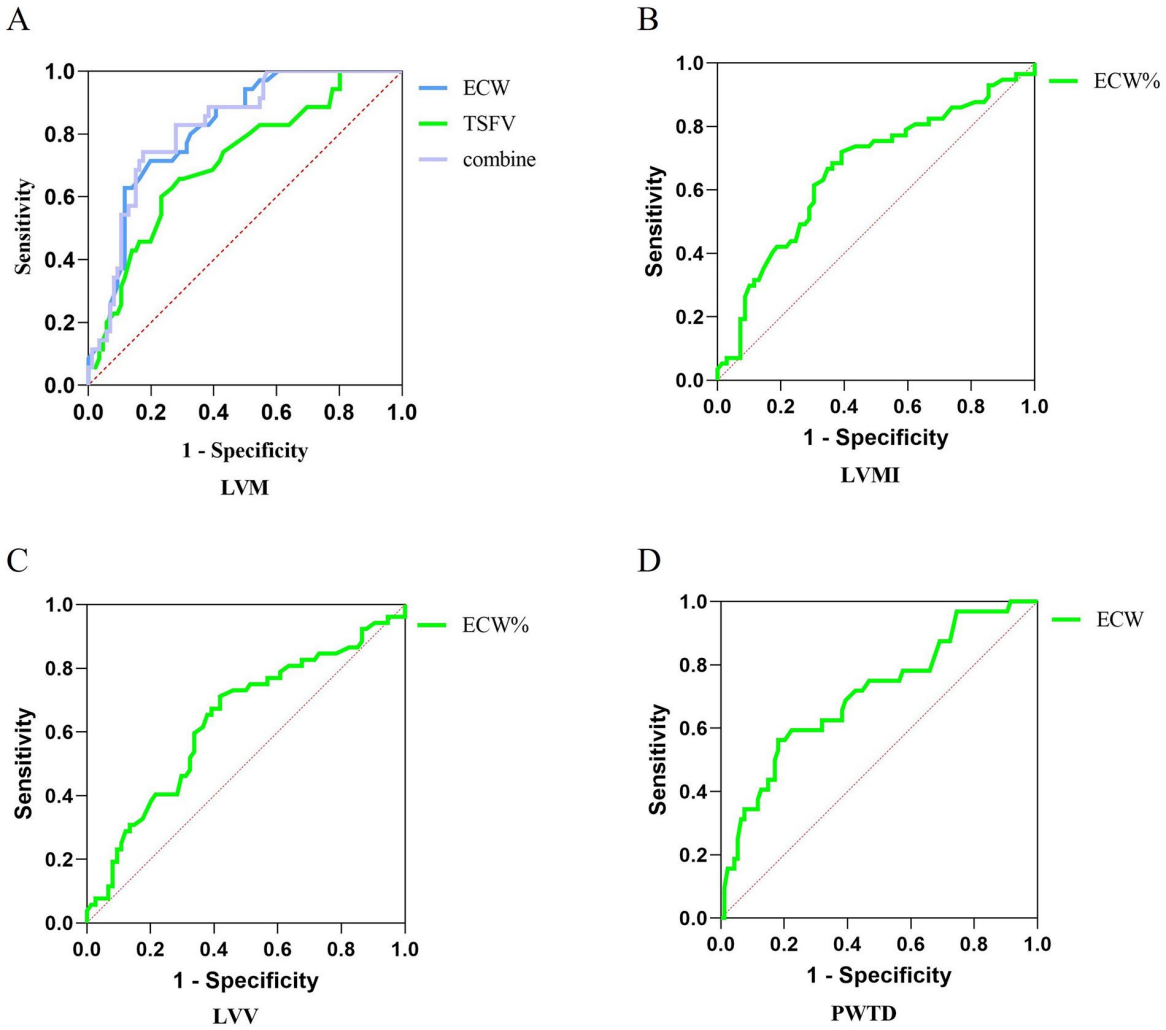
Predictive value of patients’ volume indices for cardiac function. 126 patients were taken to ROC curve, Predictive value of ECW and TSFV for LVM(A), ECW% for LVMI(B), ECW% for LVV (C), ECW and TBW for PWTD(D). The data was analyzed using the ROC method. LVM: left ventricular mass; LVMI: left ventricular mass index; LVV: left ventricular volume; PWTD: posterior wall thickness at end-diastole; ECW: the extracellular water; ECW%: percent of extracellular water; TSFV: third interstitial fluid volume.

Based on the logistic regression findings, ROC curves indicated that ECW and TSFV had high predictive values for LVM, The area for ECW predicted LVM was 0.817 (*p* < 0.001); The area for TSFV predicted LVM was 0.713 (*p* = 0.002). The ROC curve predicting the probability of ECW combined with TSFV showed a sensitivity of 74.3% and a specificity of 82.6% (AUC = 0.823, threshold value = 0.57, *p* < 0.001). ECW% had a high predictive value for LVMI (AUC= 0.710, *p* = 0.0018). ECW also showed some predictive value for PWTD (AUC = 0.707, *p* = 0.005), but ECW% had limited accuracy in predicting LVV (AUC < 0.7, *p* > 0.05) (Figure 10).

## 4. Discussions

In the present study, we found that ECW and TSFV showed positive associations with cardiac parameters by ECG, including LVV, LAV, LVDD, and LVDS. In addition, we found that ECW% also demonstrated positive associations with LVM, LVMI, LVV, LAV, IVSTD, LVDD, and LVDS. It indicates that volume control guided by bioelectrical impedance leads to improved cardiovascular outcome, namely a significant decrease in LVMI and other cardiac parameters. Additionally, patients grouped by TBW exhibited significantly higher cardiac indices in the higher TBW group.

Compared with the general population, dialysis patients face significantly higher mortality risks, which is mainly due to cardiovascular events (CVE) and is associated with abnormal fluid status [21,22]. Prior studies have demonstrated that extending the interdialytic interval results in heightened fluid burden, thereby exacerbating cardiovascular risks among patients [23]. Moreover, volume load has been identified as a crucial determinant of intra-dialytic hypertension by BIA combined with ECG [14,24], which is common in hemodialysis patients and is a crucial mediator of cardiovascular morbidity and mortality in this population.

BIA derives fluid status relative to a healthy population, and body composition, such as lean and fat mass; the latter are of value for nutritional assessments. BIA is now widely used in the management of both CKD and HD patients. It shows potential as a tool for guiding volume management in dialysis patients [25]. Volume load in dialysis patients can be reduced using the multi-frequency bioelectrical impedance, resulting in a notable drop in average stroke blood pressure and improved overall blood pressure [13]. Echocardiography is one of the most well-known methods for the assessment of the volume status in HD patients. It can provide detailed images of the heart’s structure and function, and estimate the volume status and guide fluid removal during dialysis by evaluating changes in cardiac function parameters [26].

Echocardiography is available in most centers and BIA is proposed as the preferred technique for determining the volume in HD patients. Due to the ECG requirements of professional ultrasound technicians and the restrictions on bedside access of dialysis patients, it is not very convenient. Therefore, it is particularly important to study the relationship between the measured volume index by BIA and cardiac function parameters by ECG.

Han et al. [27] found that OH/ECW, which indicates relative fluid overload, was positively associated with LA dimension, LAVI, and E/A ratio. While OH/ECW was not significantly associated with echocardiographic values such as LVEDD, LVEDV, LVMI, and LVEF. Siriopol et al. [17] found that the ultrasonic pulmonary hyperemia score before dialysis was significantly correlated with all bioimpedance parameters. Our study found significant differences in the levels of LVV, LAV, LVDD, PWTD, LVDs, and IVSTs between tertile groups based on ECW. Specifically, the third tertile group (ECW > 16.7 L) had significantly greater values than those in the first tertile group (ECW ≤ 14.2 L). Similarly, when patients were grouped into quartiles based on ECW%, the fourth quartile group (ECW% > 27.1%) exhibited significantly higher values of LVM, LVMI, LVV, LAV, LVDD, and LVDs compared to those in the first quartile group (ECW% ≤ 23.175%). Additionally, grouping based on TSFV showed that the third tertile group (TSFV > 3.967 L) had significantly greater values of LVM, LVMI, LVV, LAV, IVSTD, LVDD, PWTD, IVSTs, and PWTs than those in the first tertile group (TSFV ≤ 2.933 L). Furthermore, when grouped into tertiles based on TBW, the third tertile group (TBW > 34.3 L) also had significantly greater values of LVM, LVV, LVDD, PWTD, IVSTs, and LVDs compared to the first tertile group (TBW ≤ 27.9 L). Our research results are basically similar to those of previous studies, and prove that there is a close relationship between volume index by BIA and cardiac function parameters by ECG [28].

Left ventricular hypertrophy (LVH) is a common structural abnormality among dialysis patients [29,30], and the prevalence of LVH reaches 70%–80% in dialysis patients [31]. In our study, both systolic blood pressure (SBP) and diastolic blood pressure (DBP) were significantly elevated in the LVH group compared to the non-LVH group. This finding suggests a close association between hypertension and LVH. Moreover, LVH was associated with higher ECW% and TBW% in this study, and indicates that chronic volume loading is one of the critical factors affecting cardiac hypertrophy, which is consistent with previous results [32,33]. Strict volume control has been shown to reduce the risk of cardiovascular death and improve LVH [34]. Assessment of fluid with bioimpedance spectroscopy provides better management of fluid status, which leads to significant improvement in the cardiovascular status of hemodialysis patients.

Multifactor logistic regression analysis using bioelectrical impedance indices such as ECW, ECW%, and TSFV as independent variables, and LVM, LVMI, LVV, and PWTD as dependent variables. The findings revealed ECW and TSFV as independent risk factors for LVM, while ECW% emerged as a significant standalone risk factor for LVMI and LVV. Moreover, ECW was identified as a notable risk factor for PWTD. These results emphasize the promising utility of bioelectrical impedance measurements in assessing cardiac function metrics, particularly volume metrics.

Another noteworthy aspect is the predictive value of ECW and TSFV for LVM, as evidenced by high areas under the ROC curve. These findings highlight the potential clinical utility of bioelectrical impedance measurement volume indices in predicting cardiac abnormalities. Nevertheless, it is important to acknowledge that the predictive utility of bioelectrical impedance indices for other cardiac functional indicators is limited.

While the study provides valuable insights, some limitations need consideration. The single-center trial with a relatively small number of patients may impact the generalizability of outcomes. Additionally, the cross-sectional nature of the study and the lack of dynamic monitoring of cardiac parameters following volume management are limitations. Future multicenter prospective studies with larger sample sizes are warranted to validate and clarify the relationship between bioelectrical impedance measurements and cardiac function in dialysis patients.

## 5. Conclusion

In conclusion, this cross-sectional study reveals a correlation between measured volume indices of bioelectrical impedance and heart function indices in dialysis patients. ECW and TSFV demonstrate positive associations with various cardiac indices, and the volume indices measured by bioimpedance analysis demonstrate predictive value for LVM and LVMI, which could potentially contribute to the prevention of cardiovascular events in dialysis patients.

## Supplementary Material

figure 2.jpg

figure 6.jpg

figure 9.jpg

figure 3.jpg

figure 7.jpg

figure 8.jpg

figure 1.jpg

figure 4.jpg

figure 10.jpg

figure 5.jpg
