# Supplementary figures and images for: Impact of volume indices in bioelectrical impedance measurement on the assessment of cardiac function indices by echocardiography in hemodialysis patients
Source: Ren Fail. 2024 Jul 8;46(2):2375103. doi: 10.1080/0886022X.2024.2375103 (PMC11232648; doi:10.1080/0886022X.2024.2375103)

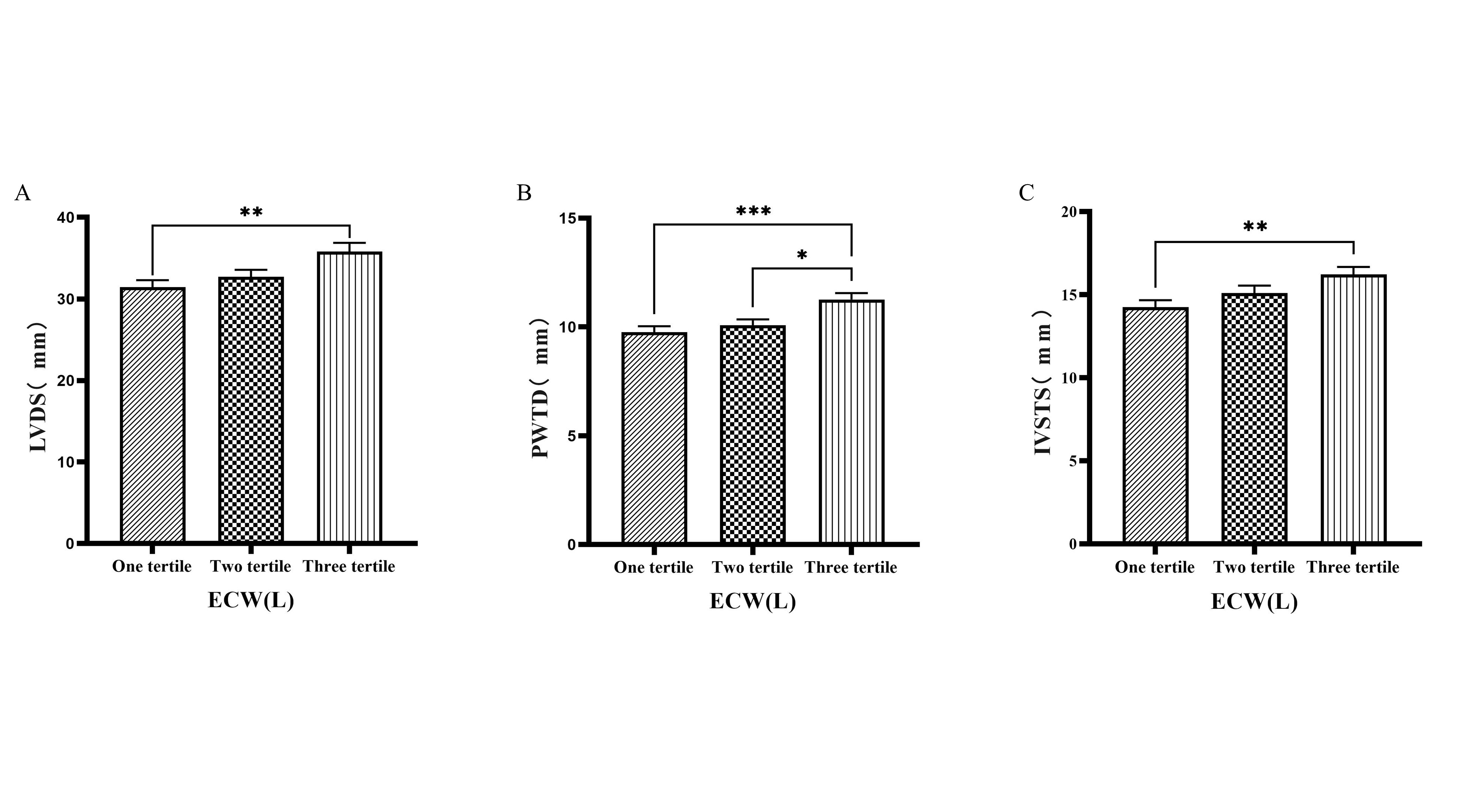

Supplement: figure 2.jpg [file IRNF_A_2375103_SM7008.jpg]

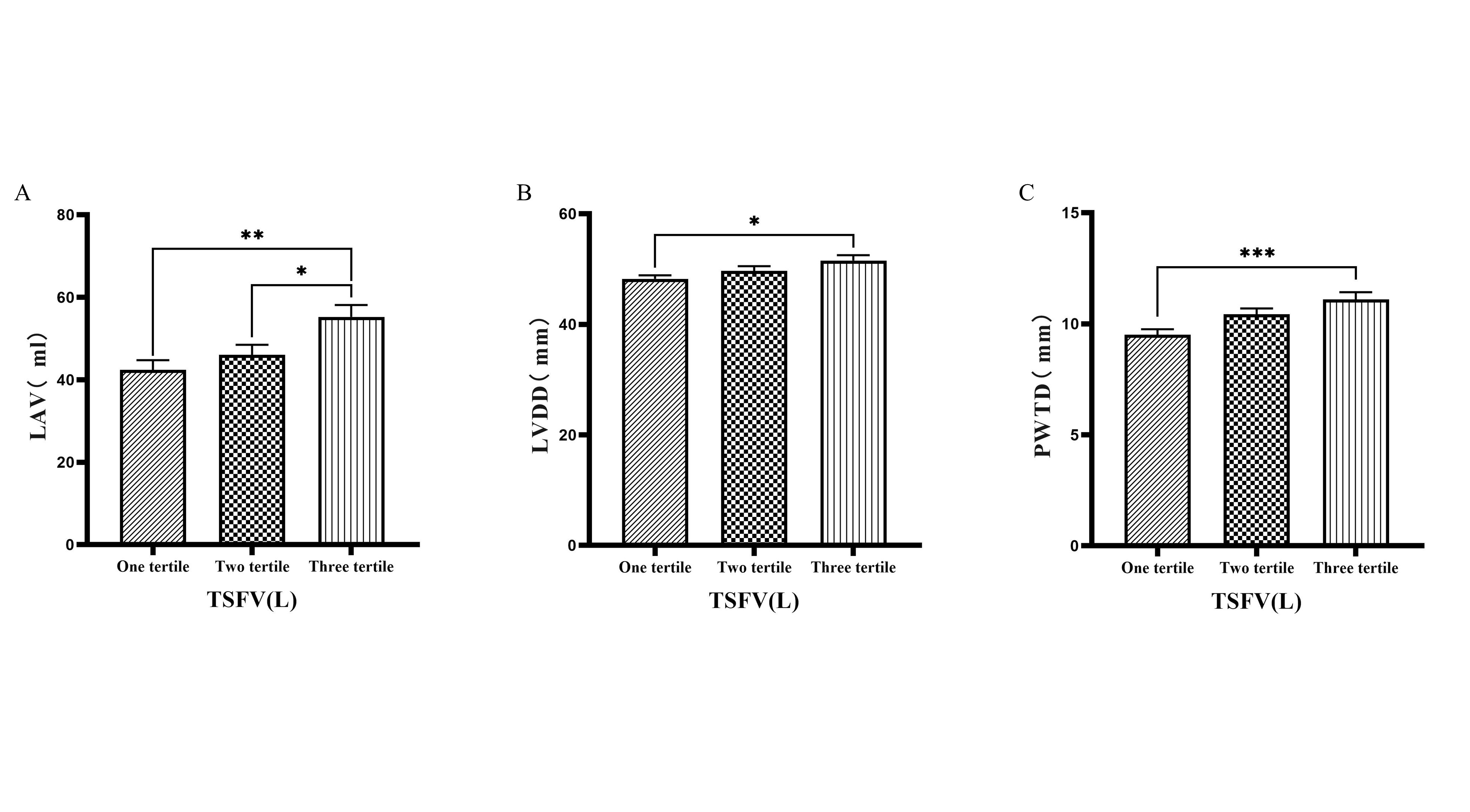

Supplement: figure 6.jpg [file IRNF_A_2375103_SM7007.jpg]

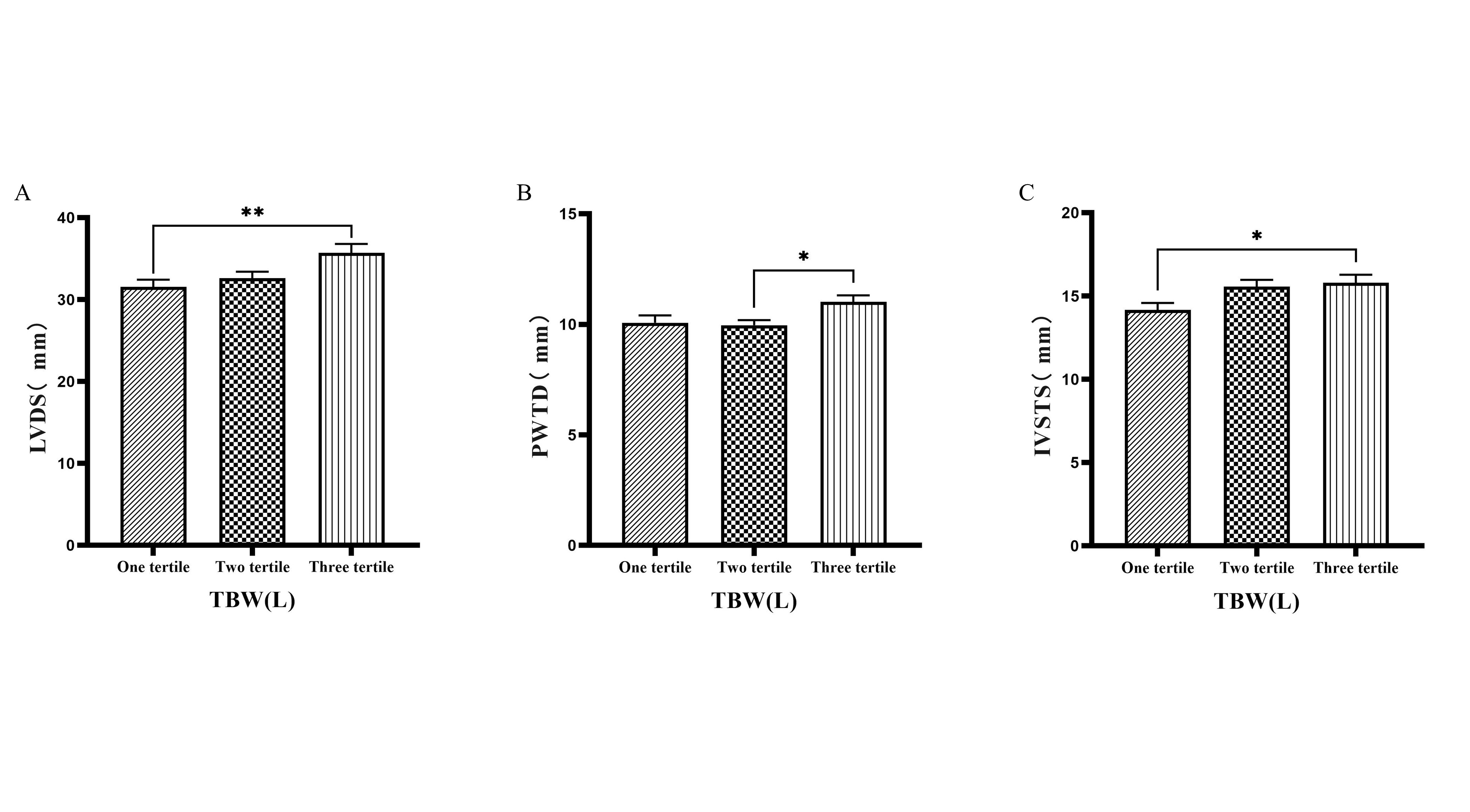

Supplement: figure 9.jpg [file IRNF_A_2375103_SM7006.jpg]

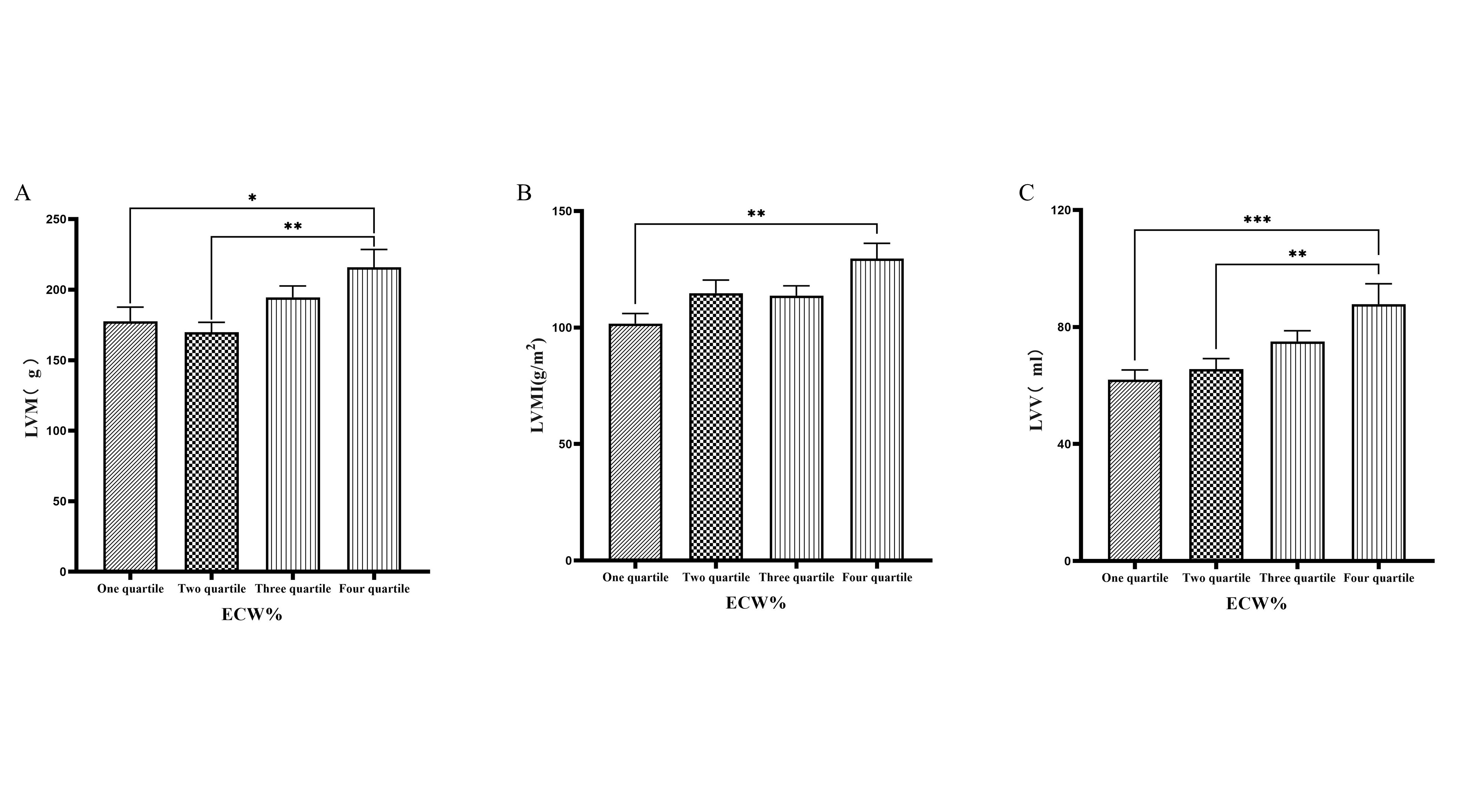

Supplement: figure 3.jpg [file IRNF_A_2375103_SM7005.jpg]

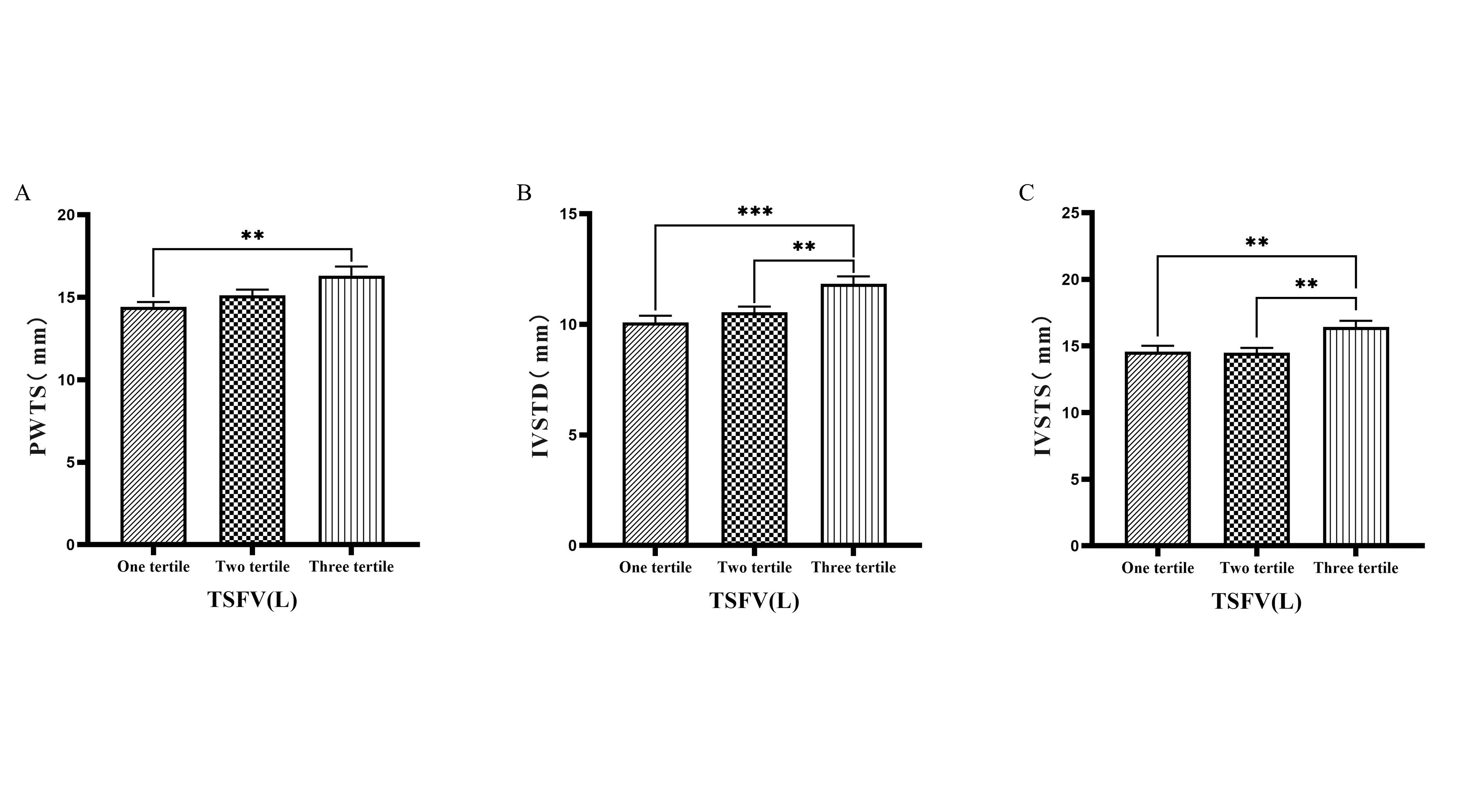

Supplement: figure 7.jpg [file IRNF_A_2375103_SM7004.jpg]

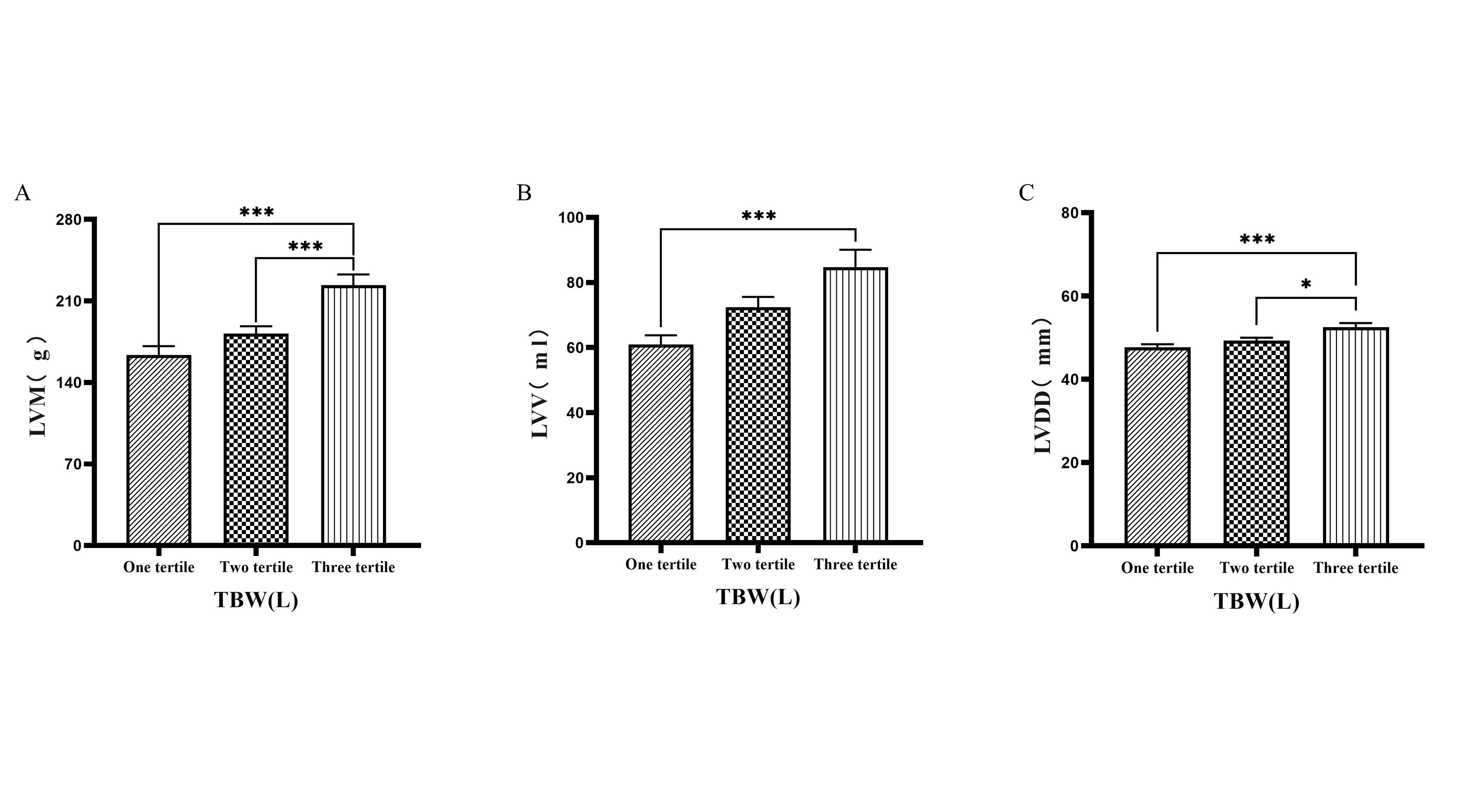

Supplement: figure 8.jpg [file IRNF_A_2375103_SM7003.jpg]

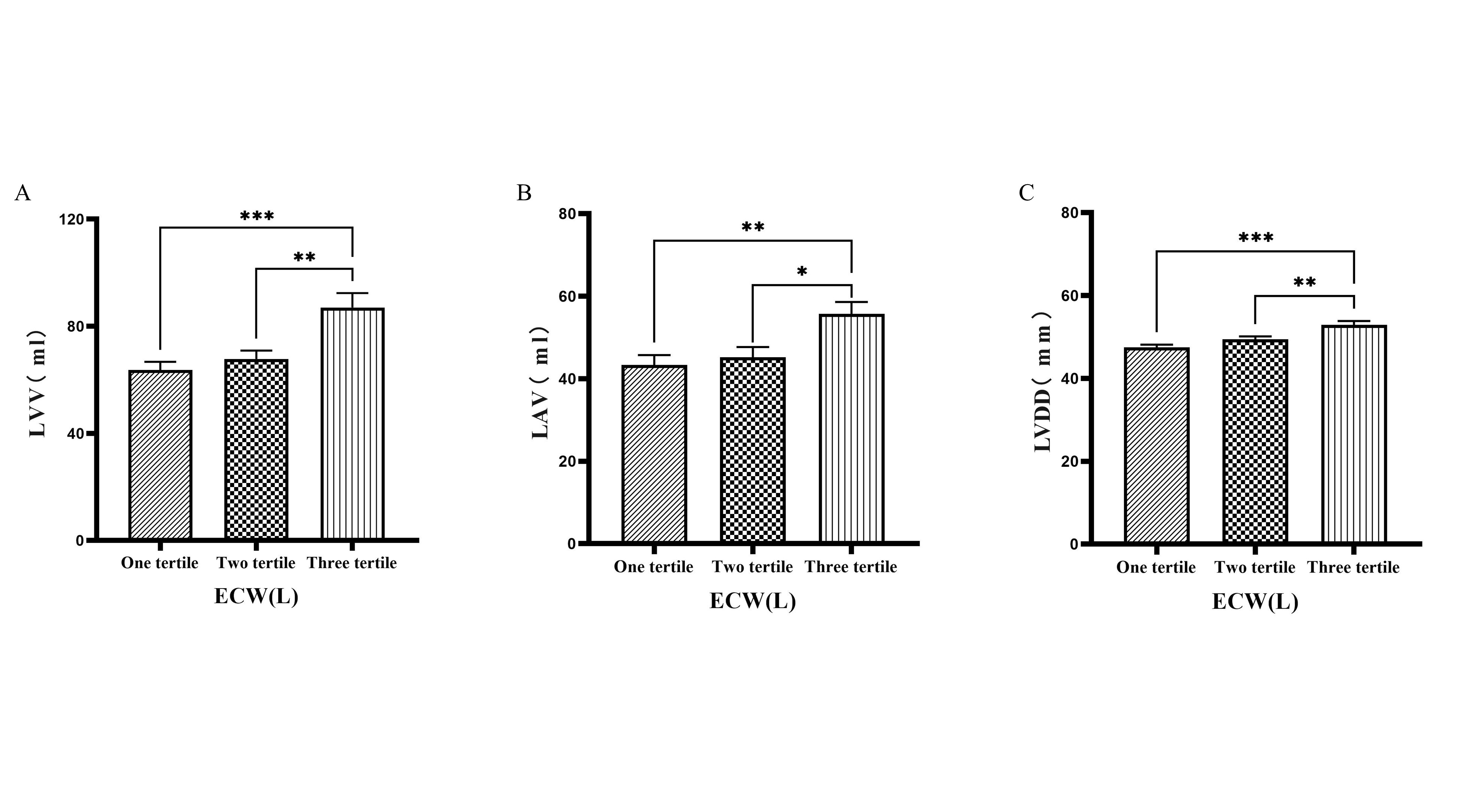

Supplement: figure 1.jpg [file IRNF_A_2375103_SM7002.jpg]

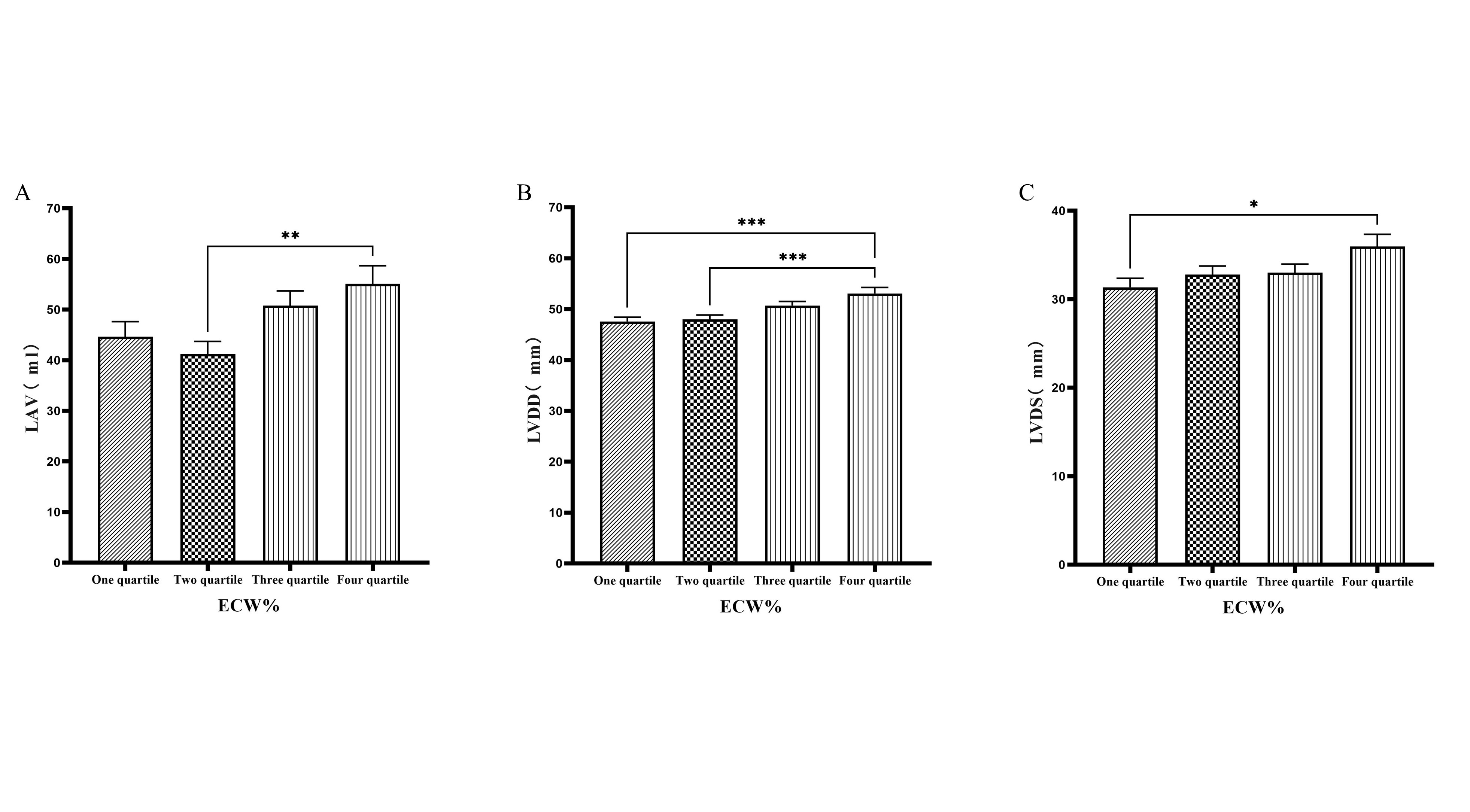

Supplement: figure 4.jpg [file IRNF_A_2375103_SM7001.jpg]

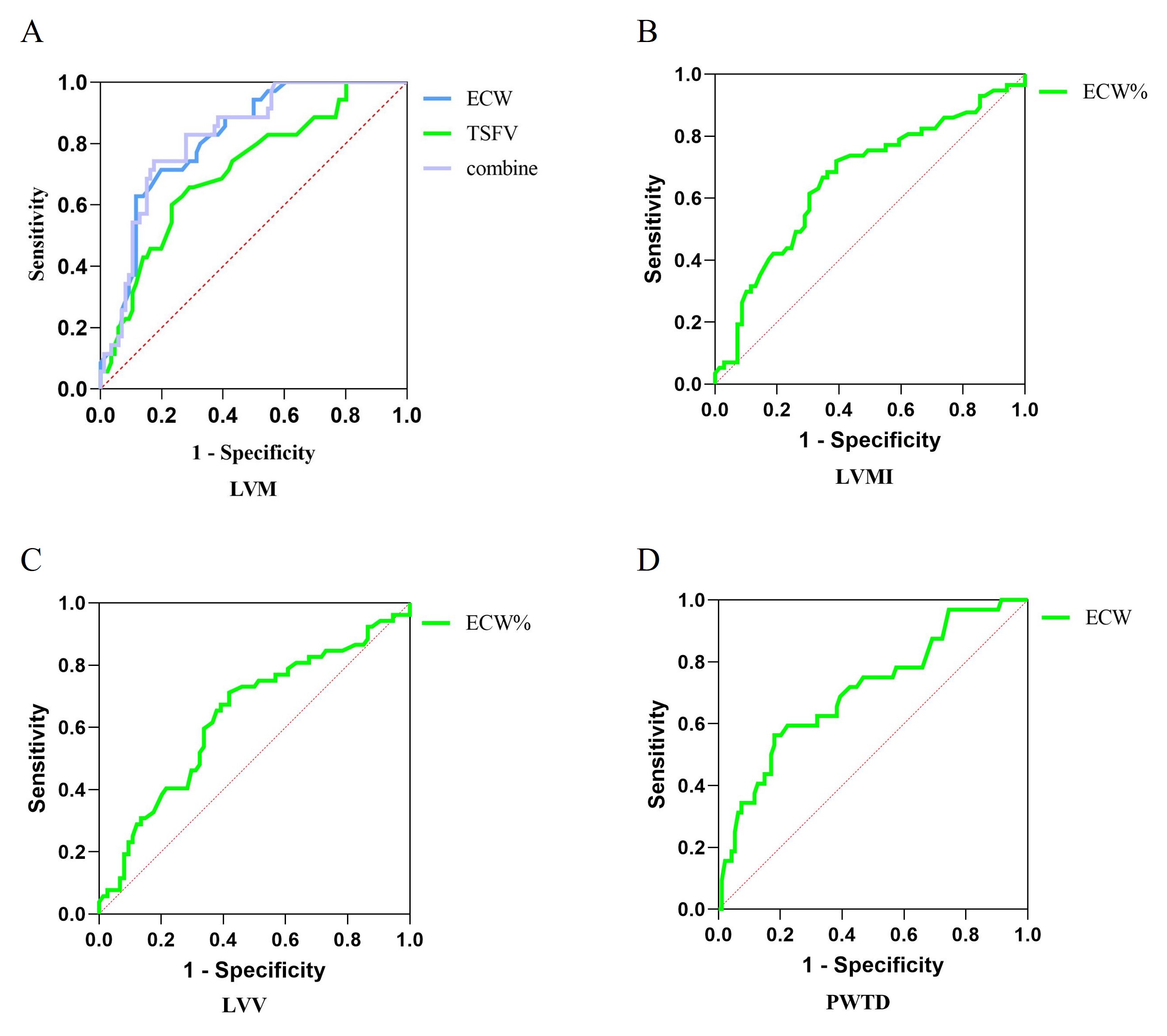

Supplement: figure 10.jpg [file IRNF_A_2375103_SM7000.jpg]

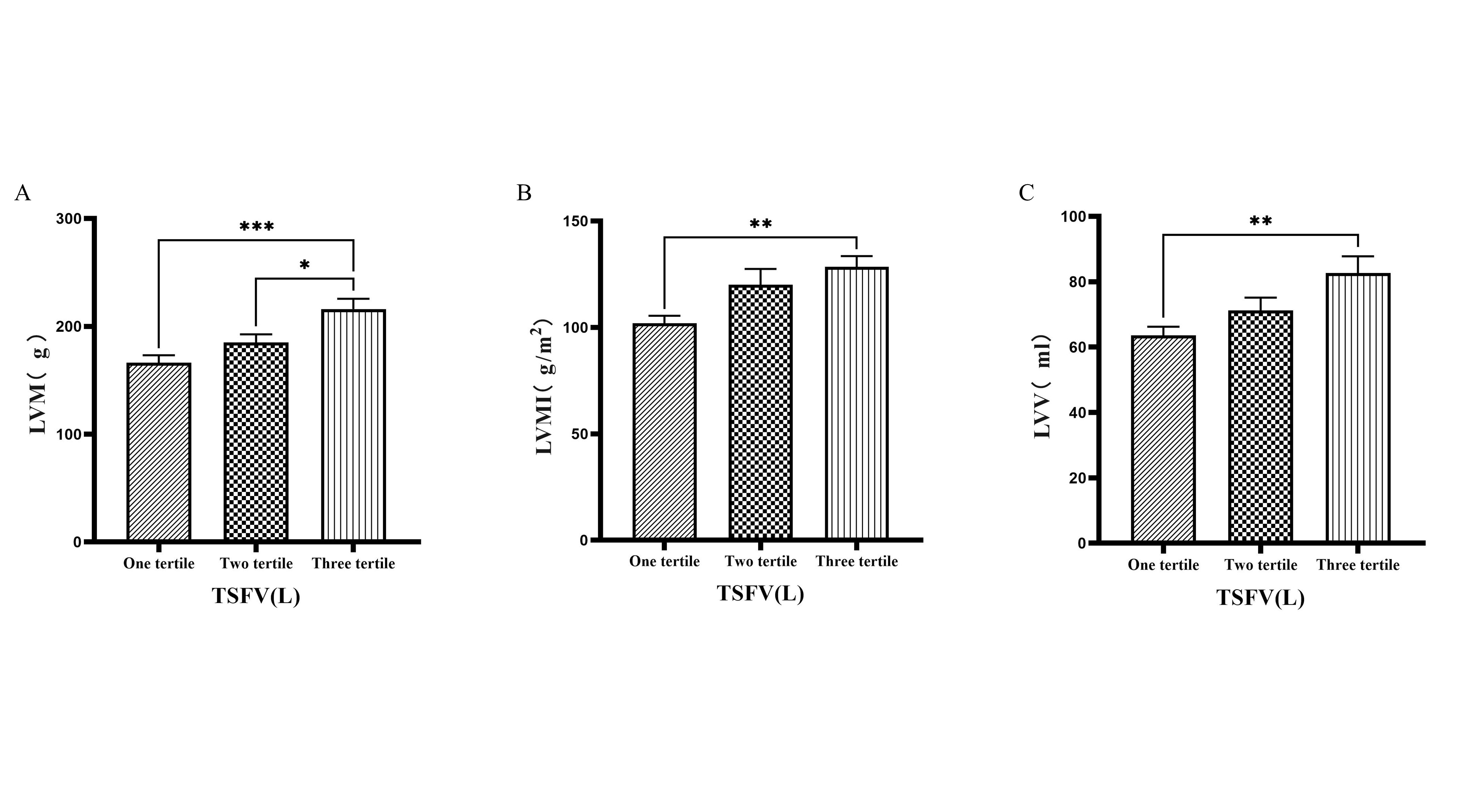

Supplement: figure 5.jpg [file IRNF_A_2375103_SM6999.jpg]
